# Supplementary material for: A closer look at the timecourse of mind wandering: Pupillary responses and behaviour
Source: PLoS One. 2020 Apr 30;15(4):e0226792. doi: 10.1371/journal.pone.0226792 (PMC7192427; doi:10.1371/journal.pone.0226792)
Supplement: Attachment — Submitted filename: Responses to reviewers.docx [file pone.0226792.s001.docx]

**Reviewer #1: summary:**
This manuscript looks at the dynamics of mind-wandering in quite a smart way: by using a task in which cues are presented that can trigger mind-wandering. Participants were asked to do a boring vigilance task, in which cue words were embedded. Whenever they noticed their mind had wandered, they we asked to press a button and report on this mind-wandering. The researchers investigated the lag between cue-words to which participants attributed their mind-wandering and the self-caught mind-wandering responses. They also examined pupil size and found it was significantly larger for mind-wandering compared to control trials.


main points
Overall I think this is a very nice study with interesting methods and results. Nevertheless, I have some questions about the methods and results.

1. The cue phrases play a major role in this method. Naively thinking, it would seem to me that the nature of these phrases makes a huge difference. How were these phrases chosen? (this would be helpful to discuss in the introduction on p.4)]

*We thank the reviewer for pointing this out. We completely agree, both the paradigm and the cue-phrases make the difference in the investigation of mind wandering.*

*We have modified the introduction to include a more detailed description and justification of our approach. We cite the numerous studies using this paradigm (with cue-phrases) to investigate different aspects of spontaneous cognition (e.g., involuntary autobiographical memories, involuntary memories and future thoughts, mind wandering). We also reference very recent TICS opinion piece by Kvavilashvili et al, (2020) specifically supporting the appropriateness of this approach for the investigation of different kinds of spontaneous cognition – in both healthy participants and in neuropsychological patients.*

### *Reference: Kvavilashvili, L., Niedzwienska, A., Gilbert, S.J., Markostamou, I. (2020).Deficits in spontaneous cognition as an early marker of Alzheimer’s disease. Trends in Cognitive Sciences. doi: 10.1016/j.tics.2020.01.005*

*In addition, the Methods section now includes more detailed information about our choice of cue-words. These come from a standardized set of 800 word-phrases, originally developed by Schlagman & Kvavilashvili (2008) with English native speakers, which we translated into Italian and adapted to Italian participants (Vannucci et al., 2015). In our adaptation study, we asked 10 independent judges to rate the level of familiarity, imageability and concreteness of the cues on a 7-point scale (1= low and 7=high). The set includes neutral, positive and negative word-phrases, thereby allowing to investigate the effect, if any, of the emotional valence of cues on the frequency and phenomenological properties of spontaneous cognition.*

2. It would be helpful to synthesize the review in the introduction the findings regarding differences and similarities between the outcomes of self-caught and probe-caught mind-wandering studies and generate hypotheses for the current study

*We thank the Editor and the reviewer for this suggestion. We synthesize these findings and clarified our hypotheses. See p. 7-8 of the revised version of the manuscript.*

3. There is quite a substantial number of participants "not complying with task instructions." What do you mean with that? And is this in any way related to your reliance on self-caught thought probes?

*No, we do not think it is related. We excluded three participants due to excessive head and body movements, which obviously compromised the reliability of pupillometry recordings. Only the fourth exclusion (1 out 28) was related to the behavioral task and it was due to an unusual report by the participant, who, at the end of the experiment, report to have forgotten to stop the task in several occasions where she had thoughts/associations elicited by the verbal cues.*

Also in relation to quality of participant's report: have you analyzed the relation between ratings of concentration and boredom and task performance? Could this be used to weed out participants who have unreliable thought probe responses?

*Thank you for raising this point. We share your view that performance could in principle be an informative parameter; however, in our participants, task performance was near perfect, leaving – unfortunately – very little variance to analyze. This is highlighted in the results session (see p. 15).*

4. What is missing from the task instructions is how participants were briefed on speed vs. accuracy. This could have substantial influence on both task performance and the tendency to mind-wander.

*We specifically avoided to stress speed over accuracy or viceversa. We had written instructions given to all participants, which simply stated that “the computer will record automatically your response and the time needed to give it”. This is now specified in the methods section.*

5. Why did you define mind-wandering episodes as 0-2 trials after the trigger or 1-3 trials before the self-report? Doesn't this specific requirement lead to specific results on the mind-wandering durations?

*We were not clear on this point, thank you for raising the issue. This window is *only* used for looking at pupil traces, *not* for analyzing mind-wandering durations, meaning that the temporal window used for pupillometry has absolutely no impact on the behavioral results (mind-wandering latencies). This point has been clarified in the revised “data encoding and analysis” section (p. 14).*

*Please see the reply to your next point regarding the impact of this temporal window on pupillometry results.*

6. What proportion of mind-wandering episodes was removed by the three criteria specified on p.10?

*Out of 225 MW episodes triggered by cue-words, about half were excluded based on those criteria (leaving 107 events for the analyses in Figure 2). We are fully aware that this is a small amount of events, raising concerns of representativeness. However, we have verified that the results obtained by this stringent selection are essentially the same as those obtained by including all available data (releasing all three selection criteria and including any trial with available pupil recordings and for which a baseline could be defined).*

*This control analysis, now reported in the ms and shown in the figure below, includes a variable number of trials which depends on the variable latency of MW episodes: at delay 0, it includes 217 out of the 225 episodes; at delay 1 it includes 178 episodes (those that continued into the trial following the word-cue); at delay 2 it includes 171 episodes (those that continued into the second trial following the word-cue). The LME analysis indicates the same significant effects as found in our main (restrictive) analysis: significant interaction of the two fixed factors, type of cue and time from cue (F(1,1336) = 34.32, p < 0.000001); significant post-hoc effect of cue-type on both the first and the second trial after the cue (F(1,428) = 23.92, p < 0.000001) and the second (F(1, 430) = 39.72, p < 0.000001).*

*The same applies to the analysis reported in Figure 3. We also ran a control analysis including all MW events, independent of their duration. We selected 312 trials at delay 0, 308 at delay -1 and 303 at delay -2 and still found significant type of episode x time interaction (F (1,1762) = 8.72, p < 0.01) and significant post-hoc effects of episode type at trial -3 (F (1, 568) = 18.41, p < 0.00005), -2 (F (1,588) = 23.18, p < 0.000001) and -1 (F (1,604) = 9.19, p < 0.005).*

*We believe that our first and more restrictive analysis is preferable over this permissive one, because our original analysis allows for comparing pupil diameter over trials for which a continuous pupil recording is available (imposing equal numerosity at the three delays). However, we agree with the reviewer that this additional analysis is an important control as it shows that our selection did not result in accidentally focusing on a small set of trials with atypical pupil behavior. For this reason we have included it in our results section (p.18-19).*

7. The analysis of the content of mind-wandering episodes is very interesting. How does that compare to your own and other studies that were using probe-caught responses, and that used "natural mind-wandering" rather than cue-evoked mind-wandering? Did you examine whether past-related mind-wandering was mostly negative as previously reported by e.g., Jonathan Smallwood?

*We have run the requested analysis and globally confirmed the previously reported association between temporal focus and emotional valence of MW events. Specifically, a linear mixed-model shows a significant effect of the fixed factor temporal focus over emotional valence (F = 3.48, p < 0.05), which post-hoc tests (Tukey) reveal to be primarily explained by significantly more negative valence associated with past-oriented relative to a-temporal MW episodes.*

*As for the effect of other variables, such as the presence of cue-words and the self-catch procedure, we absolutely agree on the possibility that they may profoundly affect the phenomenological properties of MW. However, we believe that such possibility cannot be adequately tested with the present dataset. Our experiment was specifically designed for investigating the temporal dynamics of MW, not its phenomenological properties. We are currently running a new study, in which all participants will perform the vigilance task in two versions: with and without cue-words. We feel that only in the future, with these (and other) new data, shall we approach the reviewer’s question, which goes beyond the purpose of our present experiment.*

8. I am concerned about your removal of the latencies larger than 2.5 SD since this assumes that these latencies are normally distributed, and most likely they are not (the graph looks more exponential). What happens if you do not remove these longer latencies? They may in fact carry a lot of relevant information!

*We thank the reviewer for this comment. In the revised version of the ms (new Figure 1), we report and analyse all latencies with no exclusions (see p. 16-17).*

9. In the latency data, no significant effects of content of mind-wandering were found. This could arise fron two causes: there really no being a difference, or alternatively, the data being too uncertain. To determine which one, one can use a Bayes Factor, which can easily be done with the BayesFactor in R for LMEs.

*The Bayesian analysis is now included in the revised version of the manuscript (p.17). We tested for the main effect of MW content over MW latency. Specifically, we analyzed the effect of temporal aspects of MW (with four levels) with a LME model including the random effect of the variable “subject”. The base-10 logarithm of the Bayes Factors (lg10BF) associated with the model is -1.38 (where lg10BF < -0.5 imply robust evidence in support of the null hypothesis: “the model is not a good representation of the data”). Similarly, we evaluated a LME representing the effects of emotional value (with three levels) and specificity (with two levels), again with subjects as random effect; the model was associated with a lg10BF of -4.22, implying robust evidence that this model is also not a good representation of the data.*

10. Why did you use the last second from each trial in the LMEs? Why is it important to be far away from the baseline interval?

*This choice is based on the assumption that the effect of MW develops gradually over time. For such gradual effect, we expect the difference in pupil traces to be largest in the last half of the trial in Figure 2 and the first half in Figure 3, in both cases in the part of the trial that is further from the baseline window.*

11. the discussion lacks a section comparing self-caught and probe-caught mind-wandering. How do the results compare in this study, as well as previous studies in the literature?

*We addressed this point in the revised version of the discussion please see also the reply to your comment #1).*

12. mind-wandering tends to increase during the course of the task. Was there an increase in self-caught mind-wandering responses during the second part of the task? If not, maybe this would reflect a difference between cue-evoked mind-wandering and more "natural" mind-wandering.

*Once again we agree on the importance of the question but we feel that the structure of our task is not ideal for addressing it. Our vigilance task was split into two sessions, with a 5-minutes break in between, which is likely to have interrupted any temporal gradient. In addition, we did not make any attempt to vary the order of presentations across participants, implying that time is perfectly confounded with the order of cue-words in our experiment. Finally, we did not collect any index of boredom/concentration during the task, which could have helped controlling for variables that are well known to affect MW. With all these caveats in mind, we ran the requested analyses and found a trend in the opposite direction: a reduction of MW frequency over time. The figure below shows this for all MW events, the number of which decreased both across sessions (F (1, 24476) = 5.22, p < 0.05) and within each session (F (1, 24476) = 3.88; p = 0.05). The same pattern was found for the subsample of MW triggered by cue-words (effect of session, F (1, 24476) = 9.30, p < 0.005; effect of part, F (1, 24476) = 1.02, p <0.05, data not shown).*

*We do not feel comfortable about making comparison between these results and the ones reported in the literature, because the underlying experiments differ in so *many* aspects that we deem of paramount importance, including:*

1) *We used a self-caught procedure instead of probe-caught procedure. We know that meta-awareness and monitoring abilities play a crucial role in reporting the contents of the mind in the self-caught procedure, whereas they play no role in the probe-caught method in which high levels of monitoring are imposed to participants by a specific question about the contents of one’s mind. Therefore, the changes in the amount of MW reported during the task in the self-caught method may be due to a change in the level of meta-awareness/monitoring. For example, a recent study by Varao-Sousa and Kingstone (2018) on MW during a real-world lecture has shown a different time-course for self and probe-caught MW: probe-caught showed a linear trend increasing over the course of the lecture, whereas no linear trend was found for self-caught MW.*
2) *We used a paradigm with cue-words, and we know that when the environment is semantically rich, MW is also triggered by external stimuli (both task-relevant and task-irrelevant stimuli). We do not know whether and how the presence of cues and the kinds of cues presented (e.g. the emotional valence of the cues) might affect the changes in the amount of MW experienced during the task. To assess this question, we should use a within-subjects design, and ask a group of participants to run two versions of the same vigilance task, but one with words and one without words and see whether and how the changes (if any) in the amount of MW experienced during the first and second part of the task is affected (or not) by the presence of words.*
3) *In our study we focused on spontaneous MW. In some previous studies that investigated the changes of MW during the task, no distinction between spontaneous and deliberate MW has been done, making, again, very difficult to do a comparison.*

*For all these reasons, we would prefer not to include this analysis in the manuscript.*

13. why are the data only available upon acceptance? Would be good to make these available during the review phase

*The data are available in Zenodo, at the following doi:* <https://doi.org/10.5281/zenodo.3673044>

minor points

1. p.4: Is there a reason why people think that verbal cues may increase the proportion of past-related mind-wandering reports?

*Some recent studies, carried out by independent research groups have shown that the exposure to task-irrelevant verbal cues may affect the temporal orientation of MW, increasing the proportion of past-oriented MW. As explained by Plimpton et al (2015) the presence of meaningful stimuli (irrelevant cue words) might be essential for eliciting spontaneous bottom-up retrieval processes resulting in the recall of past memories. Similar findings have been reported in studies investigating the effects of task-relevant stimuli on MW. For example a very recent study by Faber and D’Mello (2018) has shown that during real-world, semantically rich task contexts, as reading an instructional task and watching a video, approximately half of the MW episodes (assessed by self-caught method) were triggered from the task-relevant stimulus and that past-oriented MW were more likely to be triggered from the stimulus, arising from “spontaneous associations with the stimulus” (p.1) than future-oriented and introspective thoughts. These associations triggered by the semantic contents of the stimuli are more likely to lead to the spontaneous retrieval of memory due to the associative nature of memory.*

*We now refer to these studies in the revised version of the manuscript (see p. 5-6).*

2. p.7 How is the pupil diameter transformed from pixels to milliseconds using this artificial pupil? Do you have a reference for that?

*Our video-based eyetracker is equipped with a metal slide with five “artificial pupils”, with diameters 4:6mm. if the slide is positioned at the approximate location of the participants eye (as set by the chin rest), pupilsize recordings can be acquired from each of the “artificial pupils”. We did so, separately, for the 4mm, the 5mm and the 6mm ones; these measurements in pixels were plotted against physical size (4:6mm) and fit with a linear function the slope and intercept of which were used to convert actual pupil measurements from pixels to millimeters. This procedure was repeated for each participant (because the position of the camera relative to the participant's eye could change slightly, implying slightly different pixel:mm functions).*

3. p.11 "One out of 133 MW reports was excluded from the analysis of pupil diameter because of inaccurate recording of time of MNW interruption and report." How is this possible given the fact that the task is administered by computer?

*The participant reported to have pressed the button on the keyboard and gave a mental content, but the computer failed to record the key-press, presumably because the participant accidentally pressed besides the key or pressed it too softly. This only happened once, for one subject, so we exclude a system failure and impute it to a human error.*

4. On p.14: "or a non-trigger (a verbal cue presented after a MW report)". How long after a MW report were such cues presented and can we be sure that participants were really not mind-wandering anymore?

*We thank the reviewer for this question. In the instructions, we told participants that they should resume the task following the self-interruption and after providing a short description of their mental contents. Moreover, every time, after the short description was provided, the experimenter told participants that they could “go back and resume the task”. This information is now reported in the revised version of the method. So, we believe that they were not mind wandering anymore right after the re-start of the task.*

*In the first analysis of pupillometry data, we studied the timecourse of pupil diameter over two trials after MW-triggers (n=107) or non-triggers (verbal cues presented after a MW report n=226). Non-triggers followed the resuming of the task (after a MW event) by 8.5 sec on average (SD = 3.22 sec). This information is now reported in the results (p.17). The following figure also shows the distribution of the temporal distances for the 226 non-triggers used for our main pupillometry analysis; this includes few cue-words that were 15s and more away from the resuming of the task after a MW event. We do not think that our participants were likely to relapse into a MW state over such short time, especially since they were specifically told to “go back and resume the task” after every interruption and given that their instructions were to “resume the task following any self-interruption”. Nevertheless, to exclude the possibility that such relapses confounded our pupillometry results, we repeated our main analysis including, as non-triggers, only cue-words that occurred after 6s or less after the resuming of the task (n= 90; mean latency from task resuming: 5.18 sec; SD =0.99 sec). In spite of the lower number of trials, the Linear-Mixed Model analysis still reveals a significant effect of cue-type, trigger and non-trigger (F(1,587) = 15.88, p = 0.00008), like in the main analysis.*

*We report these results in the revised version of the ms (see p.19). We thank again the reviewer for this helpful suggestion.*

**Reviewer #2**

The authors investigate changes in pupil diameter associated with self-caught mind wandering using a relatively new vigilance task that included intermixed task-irrelevant cue-phrases that, according to results, frequently prompted mind wandering. An interesting aspect of this working offers insights into the temporal unfolding of MW, showing that a self-report occurred, on average, about 7 s following a cue-phrase that reportedly triggered the MW episode. This time period corresponded to increases in pupil dilatation, which replicates past findings using a probe-caught method to measure mind wandering.

For the most part the paper is well written (however, see point 3 below). Although the work does not necessarily contribute novel insights, given that it is just a conceptual replication of the authors’ previous findings, the methodological approach is sound. I have a few minor suggestions for the manuscript itself that could be easily addressed in a revision.

1.) A growing body of research has discussed the temporal dynamics of mind wandering with much theoretical debate. Given that the authors operationalize MW here as task-unrelated thought but attempt to speak to the temporal unfolding of mind wandering, discussion about the Dynamic Framework of mind wandering should be included.

Christoff, K., Irving, Z. C., Fox, K. C., Spreng, R. N., & Andrews-Hanna, J. R. (2016). Mind-wandering as spontaneous thought: a dynamic framework. Nature Reviews Neuroscience, 17(11), 718.

Christoff, K., Mills, C., Andrews-Hanna, J. R., Irving, Z. C., Thompson, E., Fox, K. C., & Kam, J. W. (2018). Mind-wandering as a scientific concept: cutting through the definitional haze. Trends in cognitive sciences, 22(11), 957-959.

Seli, P., Kane, M. J., Smallwood, J., Schacter, D. L., Maillet, D., Schooler, J. W., & Smilek, D. (2018). Mind-wandering as a natural kind: A family-resemblances view. Trends in cognitive sciences, 22(6), 479-490.

Seli, P., Kane, M. J., Metzinger, T., Smallwood, J., Schacter, D. L., Maillet, D., Schooler, J. W., & Smilek, D. (2018). The family-resemblances framework for mind-wandering remains well clad. Trends in cognitive sciences, 22(11), 959-961.

*We thank the reviewer for this very useful suggestion. In the revised version of the manuscript we introduce the two main conceptual and methodological issues recently raised in the field, that is the issue of the conceptual and operational definition of MW (see also our response to point 3) and the issue of the dynamics of MW. As for the latter, we refer to both the dynamic framework proposed by Christoff et al (2016) and the process-occurrence perspective proposed by Smallwood (2013) which introduced the important conceptual distinction, addressed in our study, between the events and processes that control and prompt the initial occurrence of MW (onset) and the processes that sustain MW over time (maintenance).*

2.) The authors are inconsistent with how they describe MW. Sometimes MW is described as spontaneous and sometimes as being cue-dependent, perhaps because of the current state of the field. For instance, in the Introduction, the authors quote Klinger “spontaneous thoughts are probably triggered by cues…,” which in itself is a bit of a paradox: how could something be both spontaneous and also triggered? In the first paragraph of the Introduction, the authors write that “our attention drifts away… which mainly occurs spontaneously” but then point to research challenging this traditional view. It might be better, then, to describe mind wandering as “traditionally thought to drift” and/or “traditionally thought to occur spontaneously” before describing previous literature to the contrary. Similarly, in the first sentence of the Discussion, the authors write that “recent studies have shown that MW is a cue-dependent phenomenon,” which sounds like MW is always triggered by some sort of cue, but then the next sentence says that external and internal stimuli “might” act as triggers. Although a minor point, it would be helpful for the readers, especially for the broad audience of PLOS ONE, if the authors provide consistency throughout the piece.

*In the original version of the ms, we provided the operational definition of MW only in the method section, when we specified the criteria that we used to categorize a mental content as mind wandering. In the revised version of the ms, we briefly mention the issue of the conceptual and operational definition of MW (currently debated in the field) and we clearly describe the operational definition of MW that we used in our study (the same adopted in our previous study with pupillometry).*

*As explained in a very recent TICS opinion, published over the last week (Kvavilashvili et al. 2020), mind wandering mainly arises spontaneously and in most of the cases people are able to clearly identify the trigger of their MW, and this might external or internal. So, the fact that MW arises spontaneously does not necessarily means that it is cue-independent. Moreover, several studies on other kind of spontaneous cognition, as involuntary autobiographical memories, episodic future thinking (see the recent special issue published on this topic in Psychological research the theoretical paper on future thinking by Cole & Kvavilashvili, 2019) have clearly shown that spontaneous thoughts can be triggered by internal or external stimuli.*

*In the revised version of the ms we better explain these points (see p.3-6).*

3.) Only two decimal places are needed for reported statistics. The authors are inconsistent with this throughout the Results section.

*Thank you, the format was revised.*

4.) The second paragraph of the Discussion is poorly written and difficult to follow. I think the task-specific aspects of the MW-pupil diameter relationship could be described with more precision. I also do not agree that “it is impossible” to predict the direction of pupil modulations in a new experimental paradigm. One could look for similarities across task parameters to make predictions. Is this why the authors do not provide a justification for sample size? One should be included.

*We completely agree with the reviewer. In the revised version of the manuscript we have revised the relevant paragraph accordingly (see p.22-23). No, we do not think this exempts us from a power analysis, which we now report in our Methods section.*

5.) The following article is highly relevant to the current work:

Faber, M., & D’Mello, S. K. (2018). How the stimulus influences mind wandering in semantically rich task contexts. Cognitive research: principles and implications, 3(1), 35.

*We thank the reviewer for this suggestion. In the revised version of the manuscript we described this study and its contribution to the research field of MW (see p.6, and also p.23).*
